# IL-21 promotes the expansion of CD27^+^CD28^+^ tumor infiltrating lymphocytes with high cytotoxic potential and low collateral expansion of regulatory T cells

**DOI:** 10.1186/1479-5876-11-37

**Published:** 2013-02-12

**Authors:** Saskia JAM Santegoets, Annelies W Turksma, Megan M Suhoski, Anita GM Stam, Steve M Albelda, Erik Hooijberg, Rik J Scheper, Alfons JM van den Eertwegh, Winald R Gerritsen, Daniel J Powell, Carl H June, Tanja D de Gruijl

**Affiliations:** 1 Department of Medical Oncology, VU University Medical Center, Cancer Center Amsterdam, Amsterdam, the Netherlands; 2 Department of Pathology, VU University Medical Center, Cancer Center Amsterdam, Amsterdam, the Netherlands; 3 Abramson Family Cancer Research Institute, Department of Pathology and Laboratory Medicine, University of Pennsylvania, Philadelphia, USA

## Abstract

**Background:**

Adoptive cell transfer of tumor infiltrating lymphocytes has shown clinical efficacy in the treatment of melanoma and is now also being explored in other tumor types. Generation of sufficient numbers of effector T cells requires extensive *ex vivo* expansion, often at the cost of T cell differentiation and potency. For the past 20 years, IL-2 has been the key cytokine applied in the expansion of TIL for ACT. However, the use of IL-2 has also led to collateral expansion of regulatory T cells (Tregs) and progressive T cell differentiation, factors known to limit *in vivo* persistence and activity of transferred TIL. The use of alternative T cell growth factors is therefore warranted. Here, we have compared the effects of IL-2, -15 and −21 cytokines on the expansion and activation of TIL from single-cell suspensions of non-small cell lung cancer, ovarian cancer and melanoma.

**Methods:**

We applied the K562-based artificial APC (aAPC) platform for the direct and rapid expansion of tumor infiltrating lymphocytes isolated from primary cancer specimens. These aAPC were engineered to express the Fc-***γ*** receptor CD32 (for anti-CD3 antibody binding), the co-stimulatory molecule 4-1BBL, and to secrete either IL-2, IL-15 or IL-21 cytokine.

**Results:**

Although IL-2 aAPC induced the greatest overall TIL expansion, IL-21 aAPC induced superior expansion of CD8^+^ T cells with a CD27^+^CD28^+^ “young” phenotype and superior functional cytotoxic effector characteristics, without collateral expansion of Tregs.

**Conclusion:**

Our data rationalize the clinical application of IL-21-secreting aAPC as a standardized cell-based platform in the expansion of “young” effector TIL for ACT.

## Background

Effective cancer immunotherapy depends on high enough frequencies of tumor-specific T lymphocytes with appropriate phenotypic characteristics, homing capacities and potent effector functions [1]. Adoptive cell transfer (ACT) has been recognized as an effective approach to achieve this. ACT using naturally-occurring autologous tumor infiltrating lymphocytes (TIL) has been studied extensively in preclinical mouse models and human clinical trials, and has been shown effective in about half of ACT-treated metastatic melanoma patients [2]. To generate sufficient numbers of effector T cells for ACT, extensive *ex vivo* expansion of T cells is required. Unfortunately, expansion often occurs at the expense of T cell differentiation and potency. Indeed, it was shown that the use of minimally cultured “young” less-differentiated TIL, with longer telomeres and higher levels of the co-stimulatory molecules CD27 and CD28, is an important factor for success. “Young” TIL demonstrated better persistence and subsequent anti-tumor activity upon adoptive transfer [3-7]. To date, IL-2 has been the consummate cytokine used in the generation of TIL for adoptive transfer [8]. IL-2, which belongs to the common γ chain family of cytokines, has been shown to promote T cell activation, proliferation and survival, and to induce tumor lysis by the expanded lymphocytes [9]. Indeed, tumor regressions have been observed upon adoptive transfer of IL-2-expanded TIL [10]. However, IL-2 can also lead to activation-induced cell death (AICD), progressive differentiation (i.e. less “young” TIL) and to the induction of suppressive regulatory T cells (Tregs) [11-13], indicating that IL-2 may also have a negative impact on the induction of an effective anti-tumor response.

IL-15 and IL-21 are also common γ chain cytokines that have been described to play a role in T cell proliferation, survival and function. IL-15 is involved in the maintenance and expansion of memory CD8^+^ T cells, NK, NKT and γδ T cells [14], and IL-21 promotes the function of effector CD8^+^ T cells [15-17]. Recent studies in mice have shown that adoptively transferred T cells demonstrated superior *in vivo* persistence and tumor elimination when pre-treated with either IL-15 or IL-21 [18-21].

Current methodology for the expansion of T cells for ACT involves the expansion of TIL from small tumor fragments or biopsies by exposure to IL-2, irradiated allogeneic feeder cells and CD3 ligation via an anti-CD3 antibody, a process called rapid expansion method (REM)[22,23]. Despite its potential, REM has been shown to greatly reduce CD28 and CD27 expression on the expanded TIL, and this is associated with reduced persistence and hence limited anti-tumor activity of the infused TIL [3,24-27]. Others and we have demonstrated previously that human peripheral blood derived lymphocytes can be expanded efficiently by K562 artificial APC (aAPC) [28-30], a standardized cell-based expansion platform, and that incorporation of the co-stimulatory molecule 4-1BBL into these aAPC results in efficient expansion of human CD8^+^ T cells with sustained CD28 and CD27 surface expression [29]. Notably, aAPC also facilitate the expansion of antigen-experienced TIL for ACT therapy [31].

In this study, we have investigated and compared the effects of IL-2, IL-15 and IL-21 cytokine on the expansion, phenotype and function of TIL from NSCLC, OvCa or melanoma biopsies utilizing aAPC engineered to express the Fcγ receptor CD32 (for anti-CD3 mAb binding), the co-stimulatory molecule 4-1BBL and either IL-2, IL-15 or IL-21. Our findings demonstrate that aAPC engineered to secrete IL-21 stimulate the preferential expansion of TIL with particularly attractive traits for ACT, i.e. high rates of CD8^+^ T cells with a less differentiated “young” phenotype and superior cytotoxic effector characteristics as well as low frequencies of CD4^+^CD25^hi^FoxP3^+^ Tregs.

## Materials and methods

### Patients and sampling of tumor biopsies

Fresh tumors were collected from patients with NSCLC or OvCa patients after obtaining appropriate informed consent under Institutional Review Board-approved protocols. The tumor lesions were collected in cold, sterile RPMI 1640 medium supplemented with 20% FCS, 50 μg/ml Gentamycine sulphate (Sigma, St. Louis, MO) and 12.5 μg/ml Amphotericin B (Gibco/Invitrogen, Carlsbad, CA) (wash medium). The tumor lesions were washed three times with wash medium, after which they were cut into small pieces with a surgical blade. Next, the cut tumor was incubated at 37°C on a shaker with digestion buffer containing 0.66 mg/ml Collagenase A (Sigma) and 0.33 mg/ml DNAse I (Roche, Indianapolis, IN). After 1 hour, the digested tumor sample was pushed through a 70 μm filter, washed 2 times with PBS and used for further evaluation and culture. HLA-A2^+^ melanoma tumor samples were selected from a previous study of Active Specific Immunotherapy (ASI) with autologous whole-cell tumor vaccines, carried out at the VU Medical Center. Tumor dissociation and cryopreservation methodologies for these samples were previously reported [32].

### Generation and culturing of lentivirally transduced K562 artificial APC

The different human K562 aAPC lines were generated and cultured as described [29]. In brief, K562 cells (American Type Culture Collection, Manassas, VA) were transduced with the lentiviral vector 4-1BB-pCLPS, after which cells with high 4-1BBL expression were cloned using high-speed MOFLO sorting (Cytomation, Fort Collins, CO). Next, 4-1BBL-expressing K562 aAPC were transduced with CD32-pCLPS, CD32-IRES-IL-2-pCLPS, CD32-IRES-IL-15-pCLPS or CD32-IRES-IL-21-pCLPS to generate no cytokine, IL-2, IL-15 or IL-21-producing K562 aAPC (hereafter referred to as no cytokine, IL-2, IL-15 or IL-21 aAPC, respectively). K562 aAPCs were cultured in AIM-V (Gibco BRL/Life Technologies, Grand Island, NY) containing 3% human AB serum (Valley Biomedical, Winchester, VA), 100 U/ml penicillin G sodium, 100 μg/ml streptomycin sulphate and 2 mM L-glutamin (Glutamax; all from Gibco/Invitrogen). Cytokine production by the aAPC was confirmed by ELISA (IL-15: Genprobe, San Diego, CA and IL-21: Biolegend, San Diego, CA) or cytometric bead array (BD Biosciences, Mountain view, CA). To this end, the aAPC were cultured overnight at 500,000 cells/ml, after which the supernatants were frozen until use.

### Polyclonal stimulation and expansion of tumor infiltrating lymphocytes

T cells were cultured in RPMI 1640 medium (Gibco/Invitrogen) supplemented with 5% human AB serum, 100 U/ml penicillin G sodium, and 100 μg/ml streptomycin sulphate and 2 mM L-glutamin (complete medium). Before stimulation, no cytokine, IL-2-, IL-15- and IL-21 aAPC were lethally irradiated with 100 Gy, washed and resuspended at 0.5 × 10^6^/ml in RPMI complete medium. Next, 500,000 aAPC were added per well in a 24 well plate and loaded with 0.5 μg/ml anti-CD3 antibody (OKT3; Orthoclone, Bridgewater, NJ) for 15 minutes at room temperature. The tumor cell suspensions were resuspended at 2 × 10^6^/ml in RPMI complete medium and 1.0 ml of cells was added drop wise to the aCD3-loaded aAPCs. T cell stimulation cultures were monitored for cell volume and enumerated on a Coulter Multisizer 3 (Beckman Coulter, Fullerton, CA) every 2-3 days. Cells were re-stimulated at 8-11-day intervals, when the expansion rate reached a plateau and/or the mean lymphocyte volume decreased to pre-expansion size. After one and/or two rounds of stimulation, the expanded TIL were used for further phenotypic and functional analysis.

### Antibodies, Tetramers and Flow Cytometry

Fluorescein isothiocyanate- (FITC), phycoerythrin- (PE), peridinin chlorophyll protein-Cy5.5- (PerCP-Cy5.5), allophycocyanin- (APC), or Alexafluor-488- (AF-488)-labeled Abs directed against human CD3, CD4, CD8, CD45RA, CD45RO, CD27, CD28, CD62L, CD56, CD19, CD14, CD25 and Granzyme B (all from BD Biosciences/Pharmingen), CCR7 (R&D systems, Minneapolis, MN), FoxP3 (Biolegend), Perforin (Ebiosciences, San Diego, CA) and matched isotype control antibodies, and PE- and/or APC-labeled HLA-A2 tetramer presenting melanoma-associated epitopes GP100_154-162_, GP100_209-217_, GP100_280-288_, and MART-1_26L-35_ (all kindly provided by Dr. John Haanen, Netherlands Cancer Institute, Amsterdam, the Netherlands) were used for flowcytometric analysis. Antibody and/or tetramer staining was performed in PBS supplemented with 0.1% BSA and 0.02% Sodium-Azide for 30 minutes at 4°C or 15 min at 37°C, respectively. Stained cells were analyzed on a FACScalibur (BD Biosciences). To exclude dead cells in flow cytometric tetramer analysis, 0.5 μg/ml Propidium Iodide (ICN Biomedicals, Zoetermeer, The Netherlands) was used. The number of CD8^+^ T cells measured was at least 100,000. T cell cultures were considered tetramer positive when tetramer + cells were equal to or exceeded 20 cells per 1 × 10e5 CD8^+^ T cells (0.02%). All flow cytometry data were analyzed with Cell Quest software (BD Biosciences).

### Cytotoxicity analysis

Cytolytic activity of the expanded TIL was analyzed by flow cytometry-based re-directed cytotoxicity assay. To this end, KT32/4-1BBL aAPC were pulsed with 1 μM of CFSE (Sigma), lethally irradiated with 100 Gy, and resuspended in RPMI supplemented with 5% hAB serum (ICN Biochemicals). Next, KT32/4-1BBL aAPC were loaded with 0.5 μg/ml anti-CD3 antibody (OKT3; Orthoclone, Bridgewater, NJ) for 15 minutes at room temperature, after which they were cultured in triplicate at a 1:2 ratio with aAPC-expanded CD8^+^ T cells. The CD8^+^ T cells were isolated from expanded TIL suspensions by magnetic cell sorting via negative selection using a CD8 T cell isolation kit (Miltenyi Biotec, Bergisch Gladbach, Germany). After 20 hours, the cells were harvested, and the percentage of cell kill was determined by 7-AAD staining (BD Pharmingen). Specific killing was determined as the percentage of 7-AAD positive cells within CFSE^+^ target cells and calculated as follows: percentage experimental 7-AAD^+^ cells minus percentage CFSE^+^/7-AAD^+^ target cells in cultures without added effector T cells (i.e. spontaneous/base level cell death).

### IFN-γ ELISA for T cell function

Tumor reactivity of IL-2, IL-15 and IL-21-aAPC-expanded CD8^+^ T cells was assessed by IFN-γ secretion. To this end, unexpanded tumor cell suspension was thawed, and CD45^+^ cells were depleted by magnetic cell sorting using CD45-microbeads (Milteny Biotec; according to manufacturer’s instruction). Next, 50,000 autologous tumor cells were cultured for 20 hours with 100,000 CD8^+^ T cells in triplicate (isolated through magnetic cell sorting as described above), after which supernatants were harvested and frozen. IFN-γ levels were determined by ELISA (sensitivity 1 pg/ml) according to manufacturer's instruction (M1933, Sanquin, Amsterdam, The Netherlands). Indicated values represent the mean cytokine concentration (pg/ml) ± SD of triplicate wells.

### Statistical Analysis

Repeated measures ANOVA was used to statistical significance of differences between groups with a Tukey post test, if data followed Gaussian distribution. If the data did not pass normality test, a Friedman test was used, followed by a Dunn’s post test. Findings were considered statistically significant when p-values were < 0.05, as indicated with asterisks (* p < 0.05, ** p < 0.01), *** p < 0.001). Statistical analyses were performed using GraphPad Prism software (version 5, 2007).

## Results

### Characterization of K562 aAPC

Expression of the Fcγ receptor (CD32) and the co-stimulatory molecule 4-1BBL was confirmed by flow cytometry for the aAPC employed (data not shown). The overnight (18 h) secreted amounts of IL-2, IL-15 and IL-21 by the corresponding aAPC were determined by ELISA as 17.0, 2.0 and 11.1 ng/ml per 500,000 cells/ml respectively, whereas the “no cytokine” aAPC did not produce any of the cytokines.

### TIL suspensions

Prior to expansion, single-cell suspensions of enzymatically-digested NSCLC and OvCa tumors were characterized by flow cytometry. As shown in Additional file 1: Figure S1, the leukocyte infiltrate ranged between 24 and 89% of the total cellular fraction, with a mean of 67% for both NSCLC and OvCa tumor digests, as indicated by CD45 staining. Moreover, NSCLC and OvCa CD45^+^ leukocytes contained similar levels of CD3^+^ T cells, B lymphocytes, NK cells, monocytes and regulatory T cells. The epithelial antigen-positive tumor fraction ranged between 1.4 and 29.2% of the overall NSCLC and OvCa cell population.

### Numerical T cell expansion

**Figure 1:**
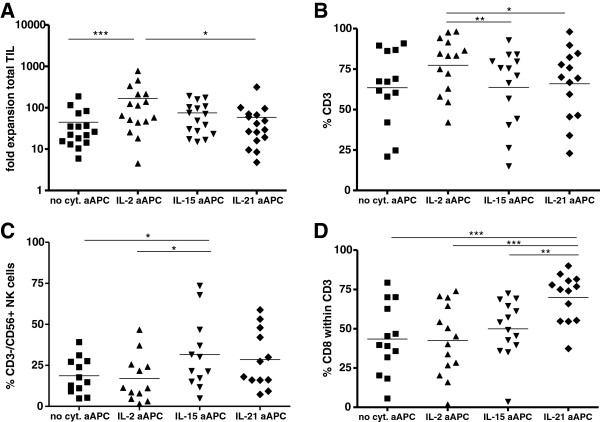
**Efficiency of tumor infiltrating lymphocytes (TIL) expansion via IL-2, IL-15, IL-21 or no cytokine aAPC.** NSCLC, OvCa and melanoma (mel)-derived TIL were expanded by stimulation with IL-2, IL-15, IL-21 or no cytokine-producing aAPC and used for further phenotypic analysis by flow cytometry after two rounds of stimulation. **A)** Fold expansion of total number of TIL cells in the IL-2, IL-15, IL-21 or no cytokine aAPC-expanded cultures is depicted for 16 NSCLC/OvCa/mel patients. **B)** Percentage of CD3^+^ T cells within the total cell population and **C)** percentage of CD3^-^CD56^+^ NK cells is shown for 12 patients. **D)** Percentage of CD8^+^ T cells within the CD3^+^ population is shown for 14 patients. Differences were compared using repeated measures ANOVA’s with a Tukey post test when data followed Gaussian distribution and by using a Friedman test with a Dunn’s post test when data did not pass the normality test. Differences were considered significant when p < 0.05, as indicated with an asterisk (* p < 0.05, ** p < 0.01), *** p < 0.001).

TIL were expanded from the tumor cell suspensions by stimulation with IL-2-, IL-15-, IL-21- or no cytokine-expressing aAPC, after which phenotype and function of the various cytokine-expanded TIL were analyzed. Comparing the numbers of T cells expanded by IL-2, IL-15-, IL-21- or no cytokine-producing aAPC revealed striking differences. As shown in Figure 1A, total cell expansion was significantly higher with IL-2 aAPC compared with no cytokine-, IL-15- or IL-21 aAPC. Furthermore, IL-2 aAPC-expanded TIL contained more CD3^+^ T cells and significantly less CD3^-^CD56^+^ NK cells as compared with IL-15 or IL-21 aAPC-expanded TIL (Figure 1B and C, respectively). Yet, IL-21 aAPC-expanded TIL contained significantly more CD8^+^ T cells as compared with IL-2-, IL-15- and no cytokine aAPC-expanded TIL (Figure 1D).

### Effect of T cell expansion on phenotype

**Figure 2:**
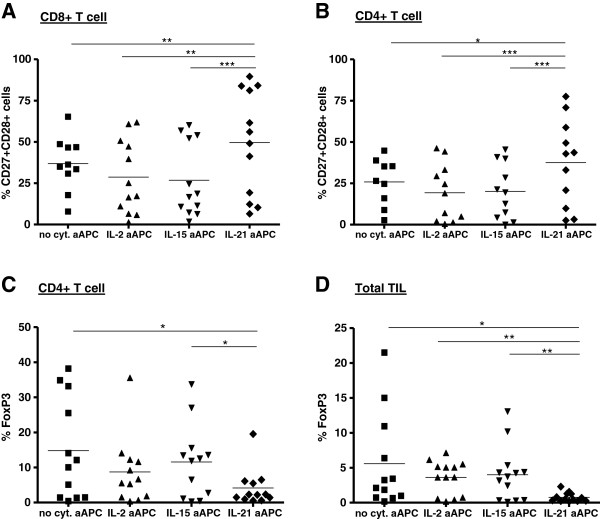
**Frequencies of CD27**^**+**^**CD28**^**+ **^**and CD4**^**+**^**FoxP3**^**+ **^**T cells in IL-2, IL-15, IL-21 or no cytokine aAPC-expanded TIL.** NSCLC/OvCa/mel-derived TIL were expanded by stimulation with IL-2, IL-15, IL-21 or no cytokine producing aAPC and used for further phenotypic and functional analysis. Percentage of CD27^+^CD28^+^ T cells was determined after two stimulations, and percentage of CD4^+^FoxP3^+^ T cells was determined after one or two stimulations by flow cytometry. Percentages of CD27^+^CD28^+^ is given for 13 NSCLC/OvCa/mel patients within **A)** the CD3^+^CD8^+^ T cell population and **B)** the CD3^+^/CD4^+^ T cell population. Percentage of suppressive FoxP3^+^ T cells is given for 13 patients **C)** as percentage of FoxP3 within CD4^+^ T cell population or **D)** as percentage of CD4^+^FoxP3^+^ cells within the total TIL population. Differences were compared using repeated measures ANOVA’s with a Tukey post test when data followed Gaussian distribution and by using a Friedman test with a Dunn’s post test when data did not pass the normality test. Differences were considered significant when p < 0.05, as indicated with an asterisk (* p < 0.05, ** p < 0.01), *** p < 0.001).

The expanded TIL were analyzed after 2 rounds of expansion for their (effector/memory) differentiation stage and the presence of CD4^+^FoxP3^+^ suppressive regulatory T cells. As shown in Figure 2A and B, IL-21 aAPC-expanded CD8^+^ and CD4^+^ T cells exhibited a “younger” less differentiated phenotype as they comprised significantly more CD27^+^CD28^+^ double positive cells as compared with IL-2, IL-15 and no cytokine aAPC-expanded TIL. Furthermore, IL-21 aAPC co-expanded less CD4^+^FoxP3^+^ cells. Both the percentage of FoxP3^+^ cells within the CD4 population (Figure 2C) and within the total TIL population (Figure 2D) from IL-21 aAPC-expanded cultures were reduced compared to all other conditions. Since activated T cells can also transiently express FoxP3, FoxP3 analysis in expanded TIL was performed when TIL returned to quiescence, i.e. when they had returned to their pre-expansion cell size, as measured by the Coulter Multisizer 3 cell sizing device.

### Impact of aAPC-based expansion on T cell function

**Figure 3:**
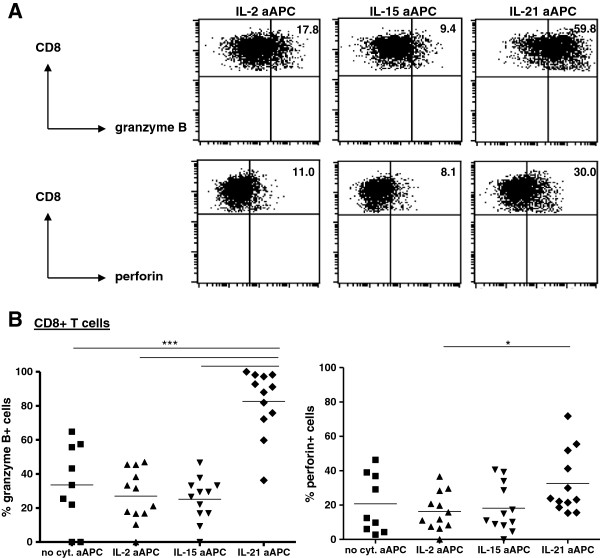
**Cytotoxic capacity of TIL expanded with IL-15, IL-21 or no cytokine producing aAPC.** Percentage of granzyme B- or perforin-expressing CD8^+^ T cells was determined by flow cytometry. **A)** Dot plots with percentages of granzyme B^+^ cells (top panel) and perforin^+^ cells (bottom panel) within the CD8^+^ T cell population are shown for a representative patient. **B)** Percentage of granzyme B^+^ cells (left) and perforin^+^ cells (right) within the CD8^+^ T cell population are shown for 12 patients. Differences were compared using repeated measures ANOVA’s with a Tukey post test when data followed Gaussian distribution and by using a Friedman test with a Dunn’s post test when data did not pass the normality test. Differences were considered significant when p < 0.05, as indicated with an asterisk (* p < 0.05, ** p < 0.01), *** p < 0.001).

**Figure 4:**
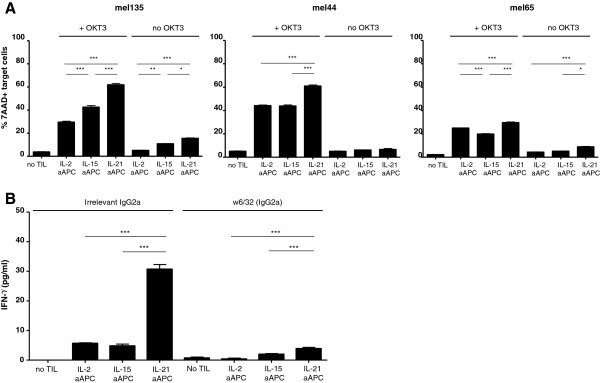
**Cytotoxic and tumor-recognizing capacity of TIL expanded with IL-15, IL-21 or no cytokine producing aAPC.** Cytotoxic capacity of the IL-2, IL-15 and IL-21 aAPC-expanded TIL was determined by flow cytometry-based re-directed cytotoxicity assay. To this end, CFSE- and anti-CD3-pulsed no cytokine aAPC cells were cultured in a 1:2 ratio in triplicate for 20 hours with CD8^+^ T cells derived from IL-2, IL-15 or IL-21 aAPC-expanded total TIL, after which percentage of killing was determined by 7-AAD staining. **A)** Data is presented as percentage of 7-AAD^+^ target cells for three patients. **B)** MHC-I restricted autologous tumor recognition of expanded TIL from one donor was determined by IFN-γ secretion assay. To this end, autologous/CD45-depleted tumor cells were cultured in a 1:2 ratio in triplicate for 20 hours with CD8^+^ T cells derived from IL-2, IL-15 or IL-21 aAPC-expanded total TIL in the presence of either irrelevant IgG2a antibody (left) or anti-MHC class I blocking antibody (w6/32; right), after which supernatants were harvested and tested by IFN-γ ELISA. Differences were compared using repeated measures ANOVA’s with a Tukey post test, and considered significant when p < 0.05, as indicated with an asterisk (* p < 0.05, ** p < 0.01), *** p < 0.001).

To assess the effects of the different cytokines during cell-based expansion on T cell function, the IL-2, IL-15 and IL-21 aAPC-expanded TIL were subjected to further functional analysis. The expanded TIL were analyzed for their cytotoxic potential by granzyme B and perforin staining. IL-21 aAPC expanded CD4^+^ (Additional file 1: Figure S2A) and CD8^+^ TIL (Figure 3) expressed significantly more perforin and granzyme B compared with the IL-2, IL-15 and no cytokine aAPC expanded TIL, suggestive of their higher cytotoxic potential. This was confirmed in an *in vitro* redirected killing assay employing anti-CD3-loaded K562 as target cells. As shown in Figure 4A, CD8^+^ T cells obtained from IL-21 aAPC-expanded melanoma TIL cultures exhibited significantly higher cytotoxic activity compared with CD8^+^ T cells obtained from IL-15 and IL-2 aAPC expanded TIL. Of note, cytotoxicity was TCR/CD3 complex-dependent, as the killing was reduced to near background levels in the absence of the CD3 antibody OKT3.

To assess whether the aAPC-expanded TIL retained tumor recognizing capabilities following expansion, TIL obtained from four HLA-A2^+^ melanoma patients were analyzed by HLA-A2 tetramer analysis. As shown in Table 1, Mart-1_26L_- and/or GP100_154/209/280_-specific CD8^+^ T cells could be detected to similar levels in fresh (i.e. unexpanded) and IL-2, IL-15 and IL-21 aAPC expanded TIL samples, indicating that tumor antigen specificity was maintained after aAPC-mediated expansion.

**Table 1:** Tetramer reactivity

**Patient**	**Epitope**	Tetramer-positivity^*^			
		**Prior to expansion**	**IL-2 aAPC expanded**	**IL-15 aAPC expanded**	**IL-21 aAPC expanded**
Mel135	MART-1-_26L-35_	**0.29**^**†**^	**0.17**	**0.20**	**0.31**
	GP100-_154-162/209-217/280-288_^‡^	n.t.	**0.03**	**0.12**	**0.13**
Mel44	MART-1-_26L-35_	n.t.	0.01	0	**0.02**
	GP100-_154-162/209-217/280-288_	n.t.	0	0	0
Mel65	MART-1-_26L-35_	n.t.	**2.23**	**2.20**	**1.14**
	GP100-_154-162/209-217/280-288_	n.t.	**0.03**	**0.03**	**0.45**
Mel84	MART-1-_26L-35_	**0.14**	**0.10**	**0.05**	**0.02**
	GP100-_154-162/209-217/280-288_	0	0.01	0	0

^*^Percentage tetramer + cells within CD8^+^ population.

^‡^Pool of 3 GP100-specific tetramers.

^†^ Response was considered positive when ≥0.02% of CD8+ T cells expressed the specific TCR (depicted in bold).

For one of these patients (mel65), viable and MHC class I expressing autologous tumor cells were available for functional testing by IFNγ secretion assay. Anti-tumor effector function of the aAPC-expanded TIL could be confirmed for this patient, as demonstrated by IFNγ secretion after co-culture with autologous tumor cells (Figure 4B). Moreover, this tumor recognition was TCR dependent, as the IFNγ secretion was reduced to background levels in the presence of an MHC class I blocking antibody. Interestingly, the IL-21 aAPC expanded CD8^+^ T cells demonstrated superior functional tumor recognition, with significantly higher IFNγ release levels than the CD8^+^ T cells expanded by IL-2 and IL-15 aAPC.

In summary, our findings demonstrate that IL-21 aAPC are preferred for the expansion of T cells for adoptive T cell transfer, as they yielded CD8^+^ T cells with superior cytotoxic effector characteristics and phenotypic traits consistent with greater *in vivo* persistence, while minimizing collateral Treg induction.

## Discussion

ACT therapy using autologous TIL or gene-modified T cells has demonstrated clinical efficacy in the treatment of cancer [2,33,34]. Generation of sufficient numbers of tumor-specific T cells with appropriate phenotype, homing capacities and sufficient effector functions are important factors for success. The current approach for the expansion of TIL for ACT involves the expansion of TIL from small tumor fragments or biopsies by exposure to high-dose IL-2, irradiated feeders and CD3 ligation via an anti-CD3 antibody (also called REM)[22,23]. Yet, REM has been shown to greatly reduce CD28 and CD27 expression of the expanded TIL, and this is associated with reduced persistence and subsequent limited anti-tumor activity of the infused TIL [24-27]. We recently showed that the aAPC platform induces numerically similar expansion of TIL compared with REM, and these TIL have a “young” CD27^+^CD28^+^ phenotype, are enriched for CD8^+^ T cells, contain fewer Tregs and are tumor-reactive [31]. In the current study, we have compared the effects of the common γ chain cytokines IL-2, IL-15, and IL-21 on the expansion and activation of TIL using this aAPC platform. Our results show that IL-21-transduced aAPC induced preferential expansion of CD8^+^ T cells from TIL, with a “young” and a superior cytotoxic phenotype and function without collateral expansion of Tregs. Notably, these favorable features were enhanced relative to those observed following the parental aAPC and exceeded those of IL-2 and IL-15 aAPC. These findings strongly support the clinical application of IL-21 aAPC in the expansion of effector TIL for ACT.

For the last 20 years, IL-2 has been the key cytokine in the generation of TIL for ACT [8]. However, IL-2 may also lead to the expansion/induction of suppressive Tregs, and this can have a detrimental effect on the anti-tumor potency of the expanded TIL. Here we found that indeed CD4^+^FoxP3^+^ T cells co-expand when making use of the aAPC platform. Interestingly, co-expansion of these CD4^+^FoxP3^+^ T cells was nearly abrogated in IL-21 aAPC-expanded TIL, suggesting a minimally suppressive TIL phenotype. The latter was in line with findings from others describing a suppressive role for IL-21 on Foxp3 expression and regulatory T cell expansion [17,35]. Of note, although FoxP3 expression is generally associated with natural Tregs (nTregs), it has also been described to be transiently upregulated in activated effector T cells, and importantly, this up-regulation has not been associated with suppressive function [36-38]. We cannot rule out that (part of) the CD4^+^FoxP3^+^ T cells are actually activated, thereby transiently expressing FoxP3. However, all analyses were performed when TIL had become quiescent and had returned to their pre-expansion size (as measured by the Coulter Multisizer 3 cell sizing device), making the relation between FoxP3 and activation less likely.

As described, one of the major problems that may limit tumor regression and durable clinical responses after ACT is the lack of persistence of TIL following infusion. Ex vivo expansion of TIL with the IL-2-based systems greatly reduces CD27 and CD28 expression, resulting in reduced persistence and subsequent limited anti-tumor activity *in vivo*[24-27,39]. In keeping with findings from others for IL-21 [18,26], IL-21 aAPCs-expanded T cells expressed significantly more CD27 and CD28, indicating that they exhibit a “younger” less differentiated phenotype, suggesting that they might persist longer *in vivo*.

Besides the maintenance and expansion of memory CD8^+^ T cells, NKT cells and γδ T cells [14], IL-15 has also been described to play an important role in the differentiation and expansion of NK cells [40-42]. This is in keeping with our observation of increased levels of CD3^-^CD56^+^ NK cells in the IL-15 aAPC-expanded TIL. The effect of IL-21 on NK cells is not completely elucidated yet. Initially, IL-21 was linked to a regulatory role in NK cell function [43], yet subsequent studies have described it as both NK activating and suppressive [44,45]. Here we found a trend towards increased frequencies of NK cells after expansion by IL-21 aAPC, suggesting that IL-21 can enhance proliferation and/or survival of NK cells. Moreover, we found that perforin expression was increased in the NK cells expanded with IL-21 aAPCs, which contrasted with NK cells expanded by IL-15 aAPC (Additional file 1: Figure S2B).

In keeping with findings by others for IL-21, expansion of TIL with IL-21 aAPC generates T cells with greater perforin and granzyme B levels and superior cytotoxic activity [46,47]. The latter is indicative of superior cytolytic and tumor recognizing capacity after transfer. Superior cytotoxic activity of the IL-21 aAPC expanded TIL was confirmed in a redirected killing assay. Although tested in only one patient, superior MHC-I restricted anti-tumor effector activity of the IL-21 aAPC expanded CD8^+^ T cells could also be confirmed by IFN-γ secretion upon exposure to autologous melanoma cells.

We demonstrated recently that tumor antigen-specific T cells were maintained after aAPC/exogenous IL-2-based expansion of TIL [31]. In line with this, comparable frequencies of MART-1-_26L_ and GP100-_154/209/280_ specific CD8^+^ T cells were detectable between fresh, and IL-2, IL-15 and IL-21 aAPC-expanded TIL. These data indicate that the use of alternative common γ-chain signaling cytokines (i.e. IL-15 or IL-21) does not significantly alter the frequency of tumor-reactive CD8^+^ T cells in the TIL suspension.

As described, the current approach for the expansion of TIL for ACT involves expansion by making use of standard REM with high-dose IL-2 [22,23]. We recently described that the level of TIL expansion achieved using aAPC with exogenous IL-2 is similar to that attained by standard REM [31]. In the current study, we could not make such comparison as standard REM was not included in our experiments. As described, standard REM has been shown to induce 500-2000-fold expansion of TIL in 14 days [3,4,23], and this is 3-10 fold higher than achieved by our IL-2-secreting aAPC. This may be explained by the lower levels of biologically active units secreted by the IL-2-aAPC, which was determined at 2200 U/ml (range 2100-2300 U/ml) by an HT2-based reporter bioassay (data not shown; [48,49]), as compared to the 6000 U/ml generally used in rapid expansion TIL cultures. In addition, differences in the preparation of the tumor cell suspensions, i.e. TIL obtained after enzymatic digestion or after 1-2 weeks culture of tumor fragments in high dose IL-2 for the aAPC and REM expansion platforms respectively, may also be of influence. Therefore, to further substantiate our findings on the suitability/applicability of the IL-21 aAPC platform for ACT therapy, a head-to-head comparative analysis between the standard REM and the IL-21 aAPC platform is warranted in further studies.

## Conclusion

In this study, we show that IL-21 promotes the expansion of CD8^+^ T cells from TIL with a less differentiated “young” phenotype, superior cytotoxic effector characteristics, and low collateral Treg numbers. We conclude that IL-21 aAPC represent a promising platform for the expansion of TIL for use in clinical ACT trials.

## Abbreviations

ACT: Adoptive cell transfer;aAPC: Artificial APC;AICD: Activation induced cell death;NSCLC: Non-small cell lung cancer;OvCA: Ovarian cancer;REM: Rapid expansion method;TIL: Tumor infiltrating lymphocyte;Treg: Regulatory T cell

## Competing interests

The authors declare that they have no competing interests.

## Authors’ contributions

SJAM designed and performed research, analyzed data and drafted and cowrote the manuscript. AWT performed research, analyzed data and drafted and cowrote the manuscript. MMS and AGMS performed research. SMA and AJMvdE treated patients and provided clinical samples. EH, RJS and WRG interpeted data and drafed the paper. DJP provided clinical samples and drafted the paper. CHJ designed research and drafted the manuscript. TDdG designed research, analyzed data and drafted and cowrote the manuscript. All authors read and approved the final manuscript.

## Supplementary Material

Additional file 1: Figure S1 Phenotypic analysis of NSCLC and OvCa tumor digests. Single cell suspensions were prepared from NSCLC and OvCa tumor samples through enzymatic digestion. Tumor digests were characterized by flowcytometry. Percentage of tumor cells (Epithelial antigen+ (Ep.Ag+)), white blood cells (CD45+), and CD3+ T cells, CD4+FoxP3+ regulatory T cells, CD3-CD56+ NK cells, CD14+ monocytes and CD19+ B cells within total cell suspension is given for **A)** 11 NSCLC samples and **B)** five OvCa samples. **Figure S2.** Cytotoxic capacity of TIL and NK cells co-expanded with IL-15, IL-21 or no cytokine producing aAPC. **A)** Percentage of granzyme B- or perforin-expressing CD4+ T cells was determined by flow cytometry. Percentage of granzyme B+ cells (left) and perforin+ cells (right) within the CD4+ T cell population is shown for 12 patients. **B)** Percentage of perforin+ cells within the CD3+CD56- NK cell population is shown for six patients. Differences were compared using repeated measures ANOVA’s with a Tukey post test when data followed Gaussian distribution and by using a Friedman test with a Dunn’s post test when data did not pass the normality test. Differences were considered significant when p<0.05, as indicated with an asterisk (* p<0.05, ** p<0.01), *** p<0.001). Click here for file
