# Supplementary material for: IL-21 promotes the expansion of CD27+CD28+ tumor infiltrating lymphocytes with high cytotoxic potential and low collateral expansion of regulatory T cells
Source: J Transl Med. 2013 Feb 12;11:37. doi: 10.1186/1479-5876-11-37 (PMC3626797; doi:10.1186/1479-5876-11-37)
Supplement: Additional file 1: Figure S1 — Phenotypic analysis of NSCLC and OvCa tumor digests. Single cell suspensions were prepared from NSCLC and OvCa tumor samples through enzymatic digestion. Tumor digests were characterized by flowcytometry. Percentage of tumor cells (Epithelial antigen+ (Ep.Ag+)), white blood cells (CD45+), and CD3+ T cells, CD4+FoxP3+ regulatory T cells, CD3-CD56+ NK cells, CD14+ monocytes and CD19+ B cells within total cell suspension is given for A) 11 NSCLC samples and B) five OvCa samples. Figure S2. Cytotoxic capacity of TIL and NK cells co-expanded with IL-15, IL-21 or no cytokine producing aAPC. A) Percentage of granzyme B- or perforin-expressing CD4+ T cells was determined by flow cytometry. Percentage of granzyme B+ cells (left) and perforin+ cells (right) within the CD4+ T cell population is shown for 12 patients. B) Percentage of perforin+ cells within the CD3+CD56- NK cell population is shown for six patients. Differences were compared using repeated measures ANOVA’s with a Tukey post test when data followed Gaussian distribution and by using a Friedman test with a Dunn’s post test when data did not pass the normality test. Differences were considered significant when p<0.05, as indicated with an asterisk (* p<0.05, ** p<0.01), *** p<0.001). [file 1479-5876-11-37-S1.pptx]

## Slide 1
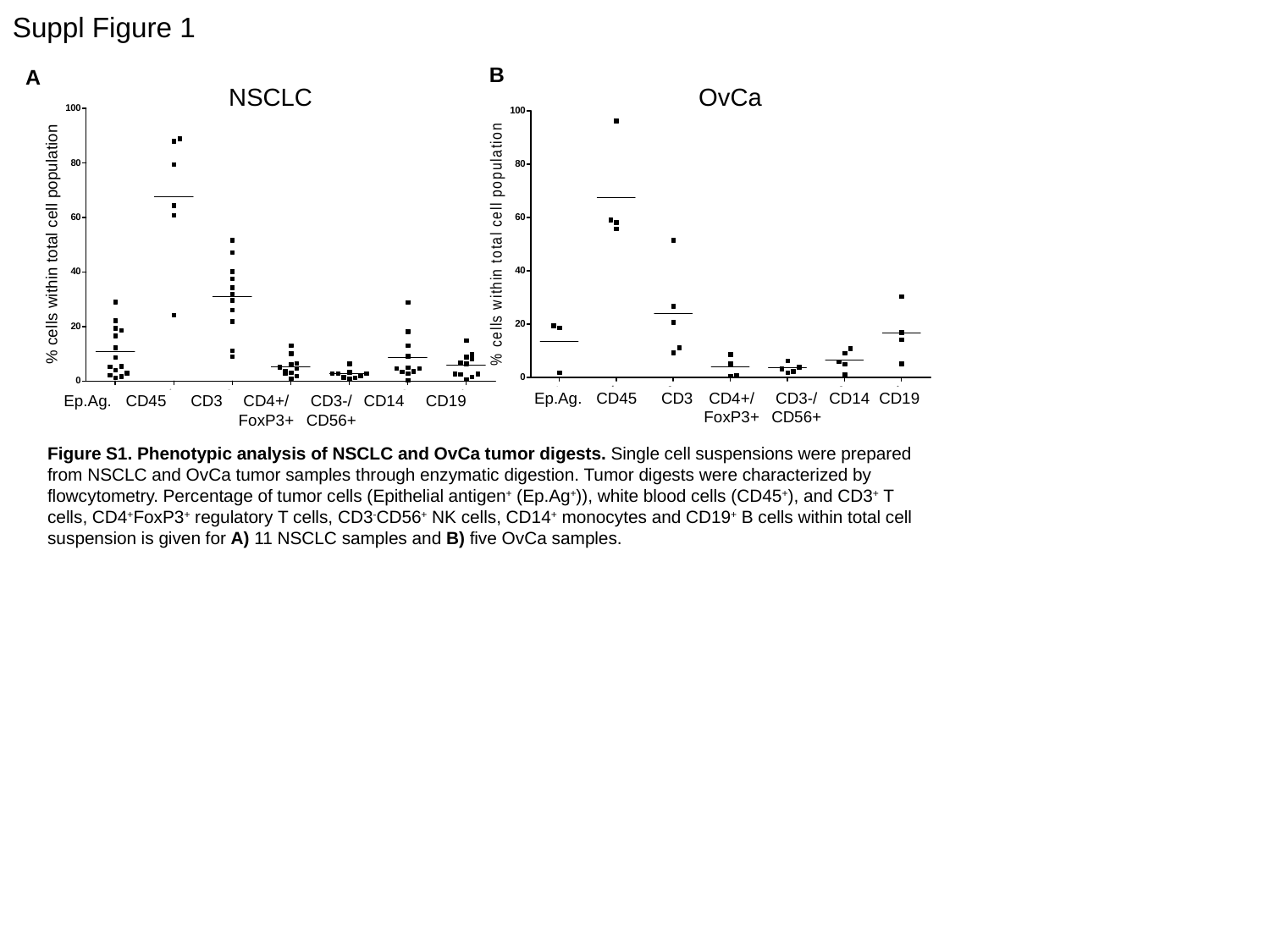

Suppl Figure 1
B
A
NSCLC
OvCa
Ep.Ag.
CD45
CD3
CD4+/
FoxP3+
CD3-/
CD56+
CD14
CD19
Ep.Ag.
CD45
CD3
CD4+/
FoxP3+
CD3-/
CD56+
CD14
CD19
Figure S1. Phenotypic analysis of NSCLC and OvCa tumor digests. Single cell suspensions were prepared from NSCLC and OvCa tumor samples through enzymatic digestion. Tumor digests were characterized by flowcytometry. Percentage of tumor cells (Epithelial antigen+ (Ep.Ag+)), white blood cells (CD45+), and CD3+ T cells, CD4+FoxP3+ regulatory T cells, CD3-CD56+ NK cells, CD14+ monocytes and CD19+ B cells within total cell suspension is given for A) 11 NSCLC samples and B) five OvCa samples.

## Slide 2
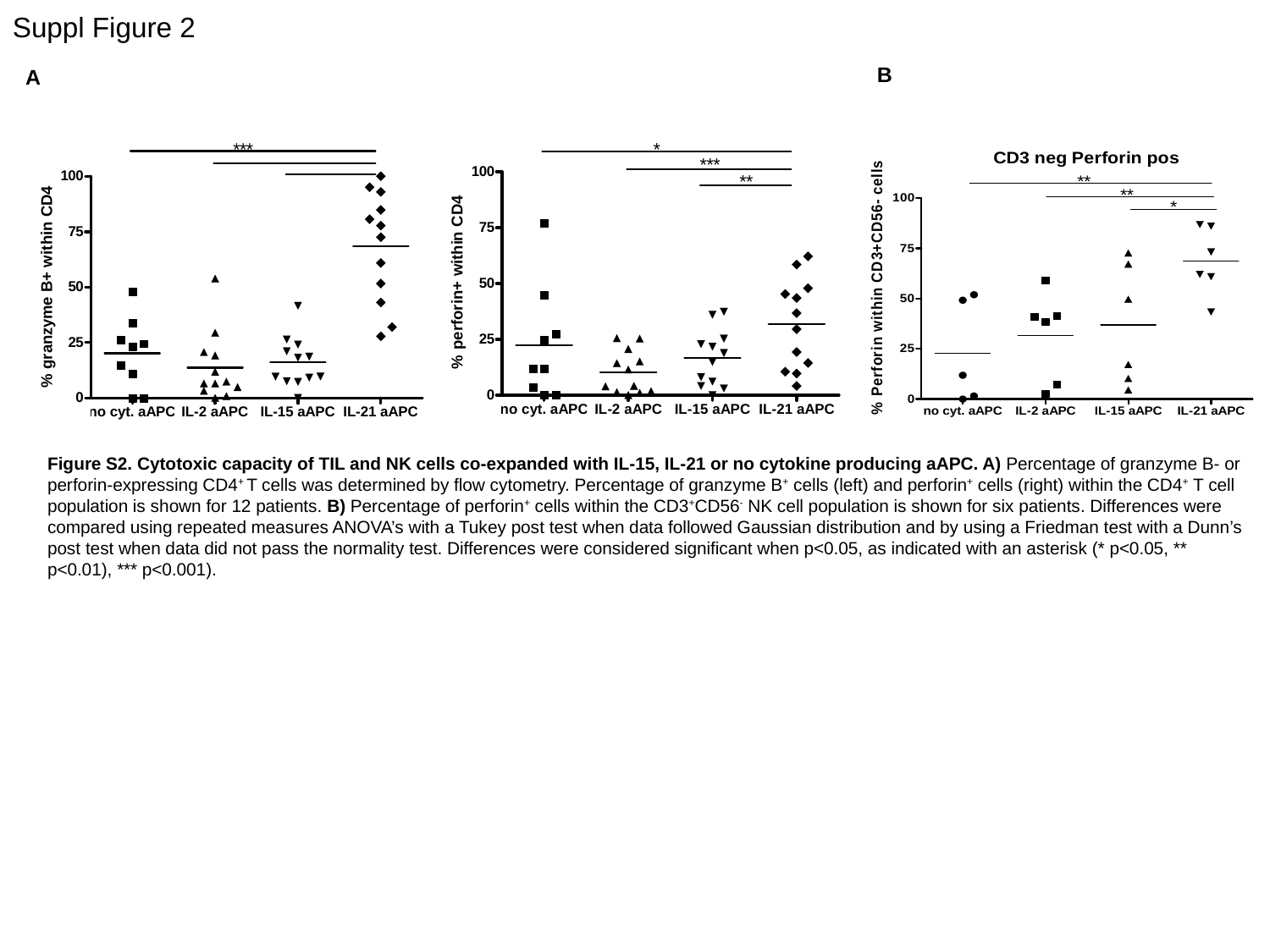

Suppl Figure 2
B
A
Figure S2. Cytotoxic capacity of TIL and NK cells co-expanded with IL-15, IL-21 or no cytokine producing aAPC. A) Percentage of granzyme B- or perforin-expressing CD4+ T cells was determined by flow cytometry. Percentage of granzyme B+ cells (left) and perforin+ cells (right) within the CD4+ T cell population is shown for 12 patients. B) Percentage of perforin+ cells within the CD3+CD56- NK cell population is shown for six patients. Differences were compared using repeated measures ANOVA’s with a Tukey post test when data followed Gaussian distribution and by using a Friedman test with a Dunn’s post test when data did not pass the normality test. Differences were considered significant when p<0.05, as indicated with an asterisk (* p<0.05, ** p<0.01), *** p<0.001).
